# Supplementary figures and images for: Fabrication of a Fish-Bone-Inspired Inorganic–Organic Composite Membrane
Source: Polymers (Basel). 2023 Oct 23;15(20):4190. doi: 10.3390/polym15204190 (PMC10611054; doi:10.3390/polym15204190)

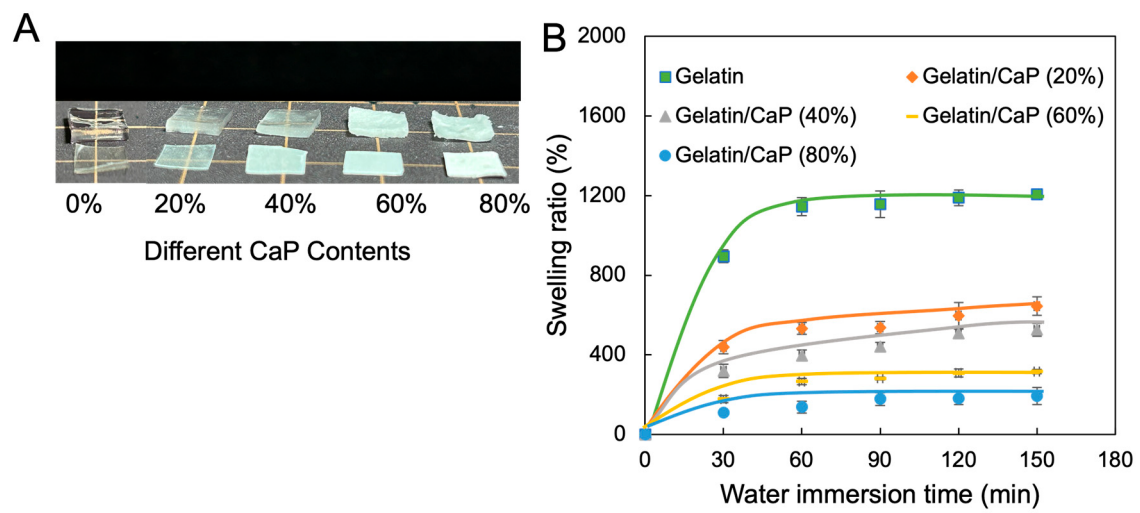

**Figure S1.** Swelling ratio of CaP–gelatin composite membrane.

Supplement: Supplementary file 1 [file polymers-15-04190-s001.zip › polymers-2640191-supplementary.pdf]
